# Use of quantitative cardiovascular magnetic resonance myocardial perfusion mapping for characterization of ischemia in patients with left internal mammary coronary artery bypass grafts

**DOI:** 10.1186/s12968-021-00763-y

**Published:** 2021-06-17

**Authors:** Andreas Seraphim, Kristopher D. Knott, Anne-Marie Beirne, Joao B. Augusto, Katia Menacho, Jessica Artico, George Joy, Rebecca Hughes, Anish N. Bhuva, Ryo Torii, Hui Xue, Thomas A. Treibel, Rhodri Davies, James C. Moon, Daniel A. Jones, Peter Kellman, Charlotte Manisty

**Affiliations:** 1 Institute of Cardiovascular Science, University College London, Gower Street, London, UK; 2 Barts Heart Centre, St Bartholomew’s Hospital, West Smithfield, London, UK; 3 William Harvey Research Institute, Queen Mary University of London, London, UK; 4 Department of Mechanical Engineering, University College London, London, UK; 5 DHHS, National Heart, Lung, and Blood Institute, National Institutes of Health, Bethesda, MD USA

**Keywords:** Perfusion, Coronary artery bypass, Grafts, Cardiovascular magnetic resonance

## Abstract

**Background:**

Quantitative myocardial perfusion mapping using cardiovascular magnetic resonance (CMR) is validated for myocardial blood flow (MBF) estimation in native vessel coronary artery disease (CAD). Following coronary artery bypass graft (CABG) surgery, perfusion defects are often detected in territories supplied by the left internal mammary artery (LIMA) graft, but their interpretation and subsequent clinical management is variable.

**Methods:**

We assessed myocardial perfusion using quantitative CMR perfusion mapping in 38 patients with prior CABG surgery, all with angiographically-proven patent LIMA grafts to the left anterior descending coronary artery (LAD) and no prior infarction in the LAD territory. Factors potentially determining MBF in the LIMA–LAD myocardial territory, including the impact of delayed contrast arrival through the LIMA graft were evaluated.

**Results:**

Perfusion defects were reported on blinded visual analysis in the LIMA–LAD territory in 27 (71%) cases, despite LIMA graft patency and no LAD infarction. Native LAD chronic total occlusion (CTO) was a strong independent predictor of stress MBF (B = − 0.41, p = 0.014) and myocardial perfusion reserve (MPR) (B = − 0.56, p = 0.005), and was associated with reduced stress MBF in the basal (1.47 vs 2.07 ml/g/min; p = 0.002) but not the apical myocardial segments (1.52 vs 1.87 ml/g/min; p = 0.057). Extending the maximum arterial time delay incorporated in the quantitative perfusion algorithm, resulted only in a small increase (3.4%) of estimated stress MBF.

**Conclusions:**

Perfusion defects are frequently detected in LIMA–LAD subtended territories post CABG despite LIMA patency. Although delayed contrast arrival through LIMA grafts causes a small underestimation of MBF, perfusion defects are likely to reflect true reductions in myocardial blood flow, largely due to proximal native LAD disease.

**Supplementary Information:**

The online version contains supplementary material available at 10.1186/s12968-021-00763-y.

## Introduction

Despite improved outcomes associated with surgical revascularisation using coronary artery bypass grafting (CABG) [1], a large proportion of patients with coronary artery disease (CAD) experience recurrent symptoms [2]. Conventional non-invasive stress test methods have reduced accuracy in identifying ischemia post CABG [3], resulting in reduced confidence in image interpretation and inconsistent impact on clinical management. Stress perfusion cardiovascular magnetic resonance (CMR) has high diagnostic accuracy for the detection of myocardial ischemia in native vessel CAD [4], but similar to other non-invasive test methods, qualitative interpretation has reduced diagnostic accuracy post CABG [5]. Quantitative perfusion mapping is being increasingly used as a fully automated, in-line tool of measuring myocardial blood flow (MBF) [6], potentially offering enhanced diagnostic accuracy and better insights into detailed distribution and severity of functional flow limitation. However, its performance in patients with left internal mammary artery (LIMA) grafts has not been previously investigated.

CABG results in significant structural and haemodynamic alterations in the heart, which makes evaluation of MBF challenging. In patients post CABG, accelerated native disease progression results in high incidence of new total occlusions in grafted vessels [7], but whether this has an impact on MBF is unclear, particularly in the presence of patent grafts. Similarly, variable extents of myocardial infarction complicate the interpretation of MBF. From a technical perspective, evaluation of MBF post CABG is also challenging. Graft length is of particular concern for any non-invasive test that relies on first pass perfusion using an intravenous contrast medium. Post CABG, the increased length of graft conduits plausibly results in a prolonged contrast transit time (arterial time delay), potentially distorting the first pass kinetics of the contrast bolus and the subsequent estimation of myocardial blood flow in graft-subtended territories [8]. This is often proposed as an explanation for the presence of perfusion defects in the LIMA territory, although evidence to support this is lacking. The deconvolution algorithm deployed for quantitative perfusion mapping includes assumptions about the maximum delay time, meaning that a true delay in contrast delivery transit time—particularly for myocardium supplied by the long LIMA graft—may result in inaccurate estimations of MBF [9].

In this study, we assess first pass perfusion CMR both qualitatively (visually) and quantitatively (perfusion mapping) in patients post CABG. By selecting a cohort of patients with angiographically-confirmed patent LIMA grafts to the left anterior descending coronary artery (LAD) and without infarction in the LIMA–LAD territory, we are able to evaluate the factors that determine myocardial blood flow in this territory, particularly the impact of native LAD total occlusion. We also evaluate the arterial time delay of contrast and assess the impact of increasing the maximal delay time programmed into the automated perfusion mapping algorithm on MBF quantification.

## Methods

This was a single centre, retrospective observational study of 38 patients with CABG undergoing stress perfusion CMR for clinical purposes between October 2017 and March 2020. All patients had coronary angiograms within 60 days of CMR. Twenty-five healthy subjects with available myocardial perfusion data were included as controls, allowing comparison of the impact of arterial delay extension on MBF estimation between patients with grafts and healthy subjects (controls) with unobstructed native coronaries. The study was approved by the National Health Service Research Ethics Committee (NHS REC) and Health Research Authority (HRA) and was conducted in accordance with the Declaration of Helsinki (REC IDs 18/LO/1583 and 19/LO/0215). All subjects provided written, informed consent.

### Study patients

Patients were included in the study if they had angiographic evidence of a patent LIMA graft to the LAD (including patent anastomosis site and distal run off), and a stress perfusion CMR either prior to or within 60 days of coronary angiography (35/38 invasive angiography, 3/38 with coronary computed tomography angiography). The clinical indication for the scans was progression of CAD and evaluation for myocardial ischemia. Patients with recent ST-elevation myocardial infarction were excluded and only patients with a LIMA graft anastomosed to the native LAD were included, allowing a focused analysis of myocardial territories with minimal variability in coronary distribution (American Heart Association (AHA) model territories 1,2,7,8,13,14) [10] (Additional file 1: Figure S1). To avoid other causes of low myocardial perfusion, patients with cardiac amyloid, hypertrophic cardiomyopathy or late gadolinium enhancement (LGE) in the LIMA–LAD myocardial segments were excluded.

### Adenosine stress perfusion CMR:

CMR studies were carried out on a 1.5T (Aera, Siemens Healthineers, Erlangen, Germany) or 3T CMR scanner (Prisma, Siemens Healthineers). Scans were performed in accordance with published recommendations [11]. Pharmacological stress was delivered with adenosine infusion at a rate of 140 mg/kg/min for 4 min with a further 2 min at 175 mg/kg/min if there was evidence of insufficient stress. Image acquisition was performed over 60 heartbeats and a bolus of 0.05 mmol/kg gadoterate meglumine (Dotarem, Guerbet, Paris, France) was administered at 4 ml/s. Basal, mid-ventricular, and apical short-axis first pass perfusion images were acquired. The sequence was then run at rest allowing measurement of rest MBF and estimation of myocardial perfusion reserve [MPR = MBF_stress_/MBF_rest_].

### Quantitative myocardial perfusion mapping

Myocardial perfusion maps were acquired using a single-bolus, dual sequence as previously described [12]. This involves the simultaneous acquisition of a low-resolution arterial input function (AIF) and a high-resolution myocardial perfusion acquisition. In-line automatic reconstruction and post processing is executed within the Gadgetron software framework [13]. MBF (ml/g/min) is calculated on a pixel-wise basis in high-resolution images using a blood tissue exchange (BTEX) model and partial differential equations which include estimation of the arterial time delay between bolus arrival in the left ventricular (LV) cavity and the pixel of interest. This is achieved by searching for the best fit function for each myocardial pixel over a period of 0 to 2.5 s, in 0.5 s steps. The arterial time delay is programmed with a 2.5 s maximum threshold as the standard default, as this has been found to cause minimal arterial time delay saturation and minimises perfusion map computation time (< 90 s) across the general patient population. To evaluate whether the 2.5 s threshold in arterial time delay resulted in saturation of estimation of arterial time delay and subsequent underestimation of MBF in patients with LIMA grafts, the myocardial perfusion maps were re-processed using a longer arterial time delay threshold up to 5 s. In a separate analysis (Additional File 1: Figure S2), the actual arterial time delay selected by the perfusion algorithm was noted, allowing comparison between CABG patients and healthy subjects.

### Image analysis

Scans were analysed visually by experienced CMR operators (attending cardiovascular imaging consultants with > 5 years CMR experience) for clinical purposes. Offline analysis was performed for evaluation of cardiac volumes, function and presence of late gadolinium enhancement using commercial software (cvi42, Circle Cardiovascular Imaging, Calgary, Alberta, Canada). First pass perfusion images were initially analysed qualitatively with a visual LIMA–LAD perfusion defect defined as an inducible perfusion defect (reduced relative signal intensity) in at least one segment from AHA model territories 1,2,7,8,13,14. Quantitative analysis of myocardial perfusion (stress, rest and calculated MPR by myocardial segment) was fully automated with no manual operator adjustment. Perfusion maps were automatically segmented using a convolutional neural network (CNN) approach with some older studies re-processed post hoc to ensure that a standardised automated analysis algorithm was used [14].

### Statistical analysis

Statistical analysis was performed in SPSS (version 26.0, Statistical Package for the Social Sciences, International Business Machines, Inc., Armonk, New York, USA). All continuous variables were tested for normal distribution (Kolmogorov–Smirnov; Shapiro–Wilk). Continuous variables are presented as mean ± SD; categorical as absolute values and percentages. Comparison of means for continuous variables was performed using a Student-t test or Mann–Whitney U test, and categorical variables were tested with χ^2^ test. Comparison of MBF between conventional (arterial time delay = 2.5 s) and increased arterial time delay (arterial time delay = 5 s) for each case was performed with Wilcoxon signed-rank test. Pre-specified variables considered likely to predict myocardial blood flow were analysed in a regression analysis. Multivariable linear regression was performed using variables significantly associated with MBF and MPR as well as important clinical and imaging variables regardless of strength of univariable associations. A bilateral p value < 0.05 was considered statistically significant.

## Results

A total of 38 patients with patent LIMA grafts and no LGE in the LIMA–LAD territory were included. Baseline characteristics including comorbidities and clinical indications for perfusion CMR are shown in Table 1. Median age was 60 years (IQR 60–73). Median time between CABG and coronary angiography was 5 years (IQR 2–11). In 30 (79%) patients, perfusion CMR was performed before coronary angiography and in 8 cases (21%) after (median 42 days, IQR 36–48). All cases were deemed to have achieved adequate stress response during perfusion.

**Table 1:** Baseline demographics and characteristics of coronary artery bypass graft (CABG) patients and healthy volunteers

	Patients with previous CABG	Healthy volunteers
Demographics	N = 38	N = 25
Age, years (median, IQR)	66 (60–73)	34 (30–43)
Sex, n (% male)	33 (87)	13 (52)
BSA, m^2^ (median, IQR)	1.9 (1.7–2.0)	2.0 (1.8–2.0)
Co-morbidities		
Diabetes, n (%)	21 (55)	
Hypertension, n (%)	37 (97)	
Hypercholesterolaemia, n (%)	33 (87)	
Medication		
B-blockers, n (%)	30 (79)	
CCB, n (%)	9 (24)	
ACE-I/ ARB, n (%)	34 (90)	
Antiplatelets, n (%)	37 (97)	
Presentation		
Typical chest pain	24 (63)	
Atypical chest pain/dyspnoea	9 (24)	
NSTEACS^a^	5 (13)	
Coronary artery bypass graft		
Time from CABG, years (median, IQR)	5 (2–11)	
Total number of grafts per patient, n (%)		
Single graft (LIMA to LAD)	1 (3)	
2× grafts	4 (10)	
3× grafts	23 (61)	
4× grafts	10 (26)	
Vein grafts per patient, n (%)		
1× vein graft	6 (16)	
2× vein grafts	21 (55)	
3× vein grafts	10 (26)	
CMR parameters		
LVEDVI, ml/m^2^	68 ± 12	77 ± 15
LVMI, g/m^2^	54 ± 13	52 ± 10
LVEF, %	62 ± 8	66 ± 4
Global stress MBF, ml/g/min	1.54 ± 0.47	2.82 ± 0.61
Global rest MBF, ml/g/min	0.82 ± 0.21	0.90 ± 0.24
Global MPR	1.94 ± 0.63	3.22 ± 0.63
LIMA–LAD (or LAD) stress MBF, ml/g/min	1.65 ± 0.54	3.04 ± 0.69
LIMA–LAD (or LAD) rest MBF, ml/g/min	0.88 ± 0.22	1.04 ± 0.30
LIMA–LAD (or LAD) MPR	1.92 ± 0.64	3.04 ± 0.65

*BSA* body surface area, *CCB* Calcium Channel blocker, *ACE-I* Angiotensin converting enzyme inhibitor, *ARB* angiotensin receptor blocker, *LVEDVI* left ventricular end-diastolic volume index, *LVMI* left ventricular mass index, *LVEF* left ventricular ejection fraction, *NSTEASCS* non-ST elevation acute coronary syndrome

^a^CMR performed for evaluation of bystander disease after coronary angiography

**Fig. 1:**
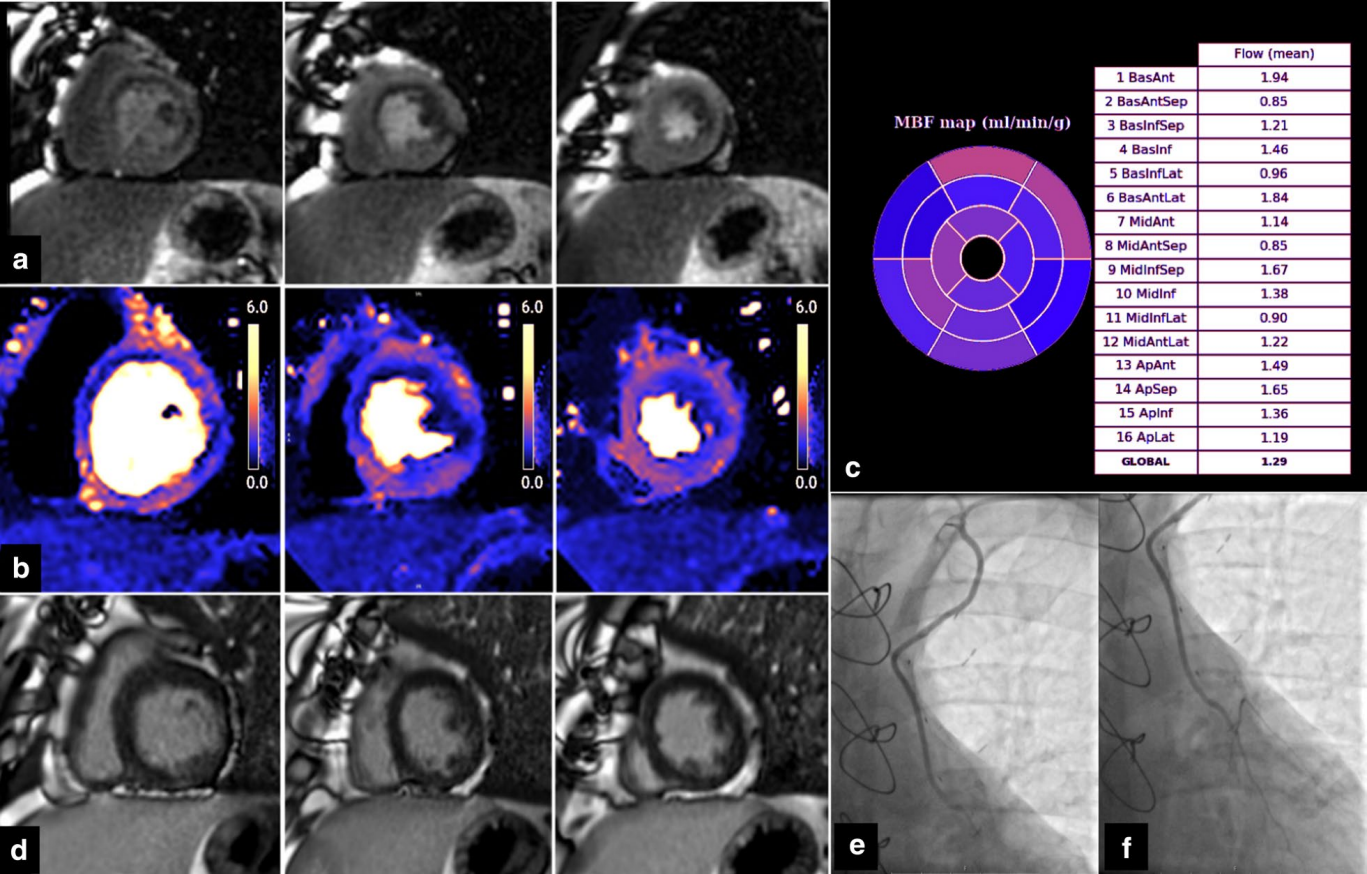
Patient with patent left internal mammary artery (LIMA)-to-left anterior descending coronary artery (LAD) and evidence of inducible perfusion defect in LIMA-native LAD territories. Short axis views from base to apex (left to right). Top row (**a**): First pass perfusion with adenosine stress, demonstrating qualitatively a perfusion defect in the basal to mid (but not apical) LAD territory. There is a second lateral perfusion defect. Middle row (**b**): Perfusion mapping showing quantitatively reduced peak myocardial blood flow (MBF) in these territories. (e.g. MBF in mid antero-septum is 0.85 ml/g/min, MBF in apical septum is 1.65 ml/g/min). **c** Bullseye plot of stress MBF in each American Heart Association (AHA) segment. Bottom row (**d**): Late gadolinium enhancement (LGE) images showing no infarction. **e**, **f** Coronary angiography demonstrating patent LIMA graft (**e**), anastomosis site (**f**) and good distal run off

By qualitative (visual) assessment, a perfusion defect in LIMA–LAD territory was reported in 27 out of 38 cases (71%) by clinical reporters blinded to the study details. This was despite all patients having patent LIMA grafts, no anastomotic stenosis, good distal LAD run off, and no LAD territory infarction (Fig. 1). Among patients with perfusion defects in the LIMA–LAD territory on visual assessment, 18 (66%) were managed medically, 1 patient had percutaneous coronary intervention (PCI) to the native LAD and 8 (30%) underwent PCI in myocardial segments not supplied by the LIMA–LAD (these patients had additional perfusion defects in other territories).

Stress myocardial blood flow in the LIMA–LAD territory was lower in patients with inducible perfusion defects on visual qualitative assessment, compared to those deemed to have no perfusion defects (1.45 ± 0.45 ml/g/min, vs 2.12 ± 0.43 ml/g/min, p < 0.001).

### Predictors of myocardial blood flow in the LIMA–LAD territory

A number of prespecified clinical and imaging variables were included in a univariable regression analysis to determine the predictors of stress MBF in the LIMA–LAD territory. These included age, sex, presence of native LAD total occlusion (proximal to the LIMA insertion), diabetes mellitus, left ventricular (LV) mass index (LVMI), LV ejection fraction (LVEF), and use of b-blockers. Amongst these, total occlusion of the native LAD proximal to the anastomosis of the LIMA (evaluated in a binary fashion; present or absent) was a strong predictor of both stress MBF (B = − 0.41, 95% CI − 0.73, − 0.009; p = 0.014) (Table 2) and MPR (B = − 0.56; 95% CI − 0.95, − 0.17; p = 0.005); (Additional file 1: Table S1) in this territory, and remained significant in multivariable analyses.

**Table 2:** Predictors of stress myocardial blood flow (stress MBF) in the LIMA–LAD territory

Independent variables	Univariate predictors			Multivariate predictors		
	B	95% CI	*p* value	B	95% CI	*p* value
Age	− 0.02	− 0.04 to (− 0.001)	**0.042**	− 0.15	− 0.03 to 0.003	0.097
Native LAD occlusion	− 0.47	− 0.79 to (− 0.15)	**0.005**	− 0.41	− 0.73 to (− 0.09)	**0.014**
LVEF	− 0.02	− 0.04 to 0.04	0.118	− 0.02	− 0.04 to (− 0.005)	**0.014**
Diabetes	− 0.18	− 0.53 to 0.18	0.320	− 0.18	− 0.49 to 0.14	0.261
LVMI	− 0.01	− 0.02 to 0.01	0.177			
Sex (Male)	− 0.31	− 0.83 to 0.21	0.236			
Beta-blockers	0.30	− 0.016 to 0.76	0.193			

LIMA–LAD territory (average of stress MBF in myocardial segments 1,2,7,8,13,14)

Bold p-values are statistically significant

### Impact of native LAD chronic total occlusion on LIMA–LAD territory perfusion

**Fig. 2:**
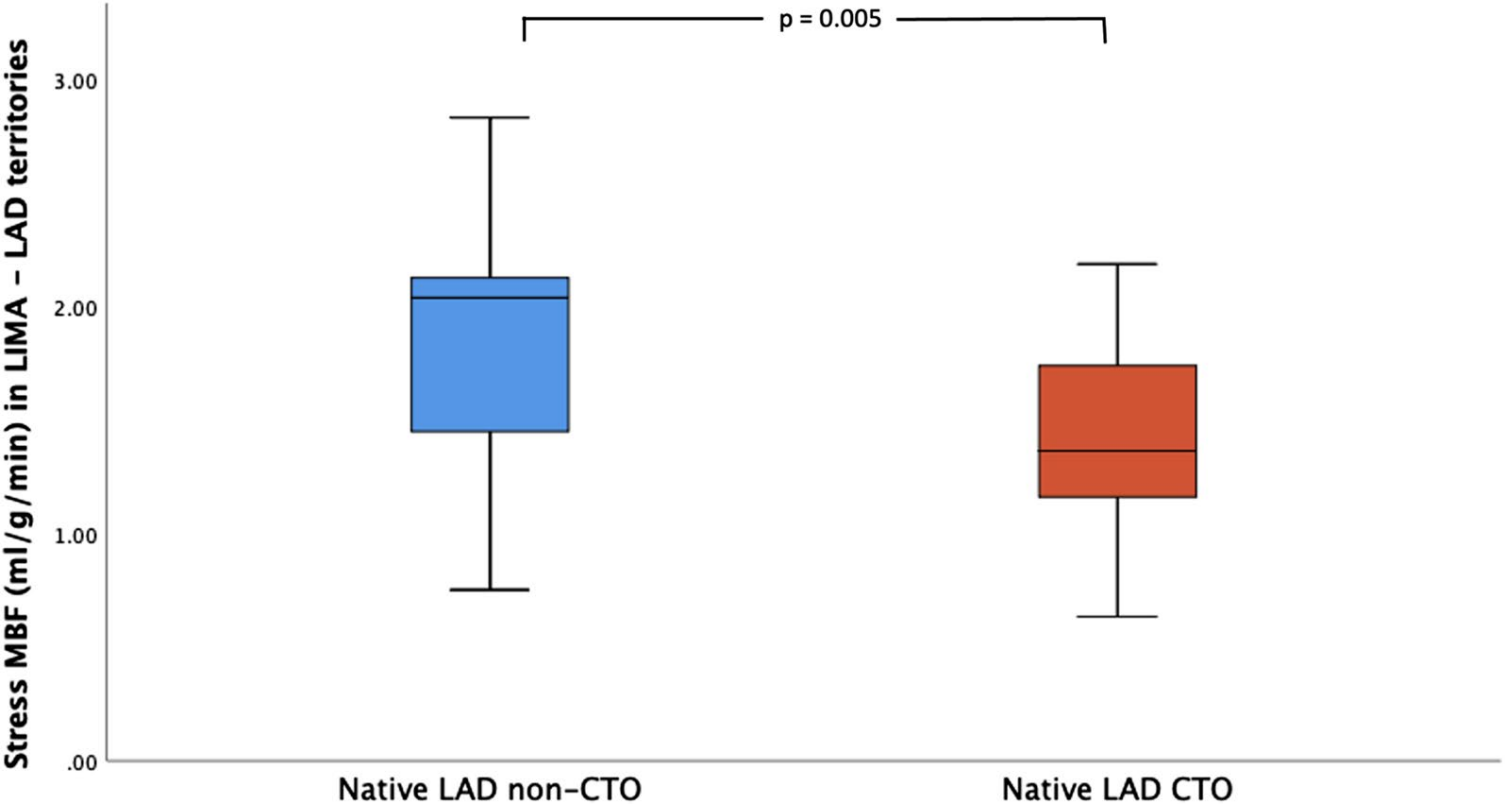
Box-plot showing stress MBF in the LIMA–LAD territory (AHA 1,2,7,8,13,14) depending on native LAD status. Total occlusion of the native LAD was associated with significant reduction in stress MBF. Error bars represent 95% CI

**Fig. 3:**
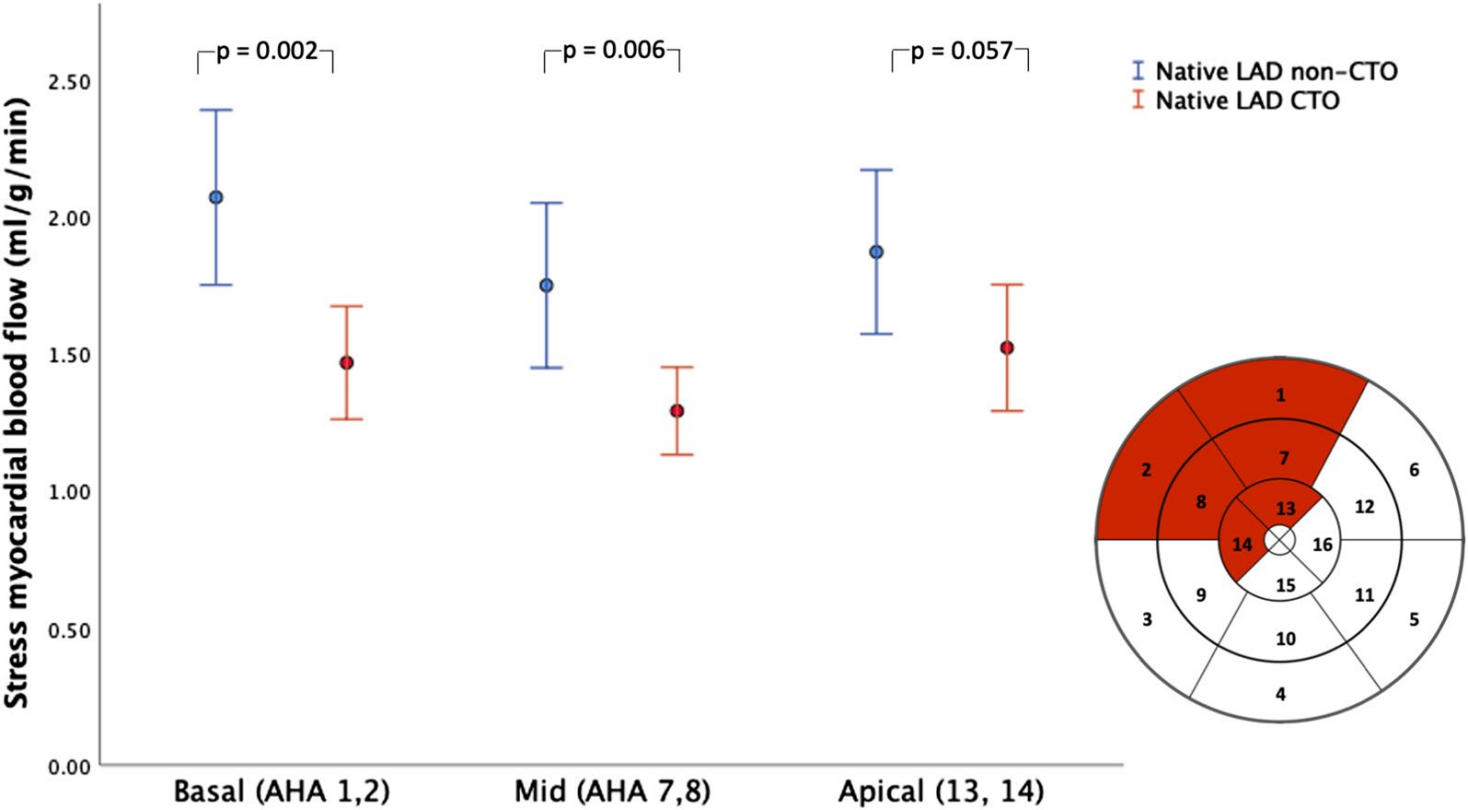
Stress MBF within the LIMA–LAD territory in each myocardial level (basal, mid, apex). Total occlusion of the native LAD was associated with a reduction in peak MBF of the basal and mid-but not apical LAD segments. Error bars represent 95% CI

Stress MBF in the LIMA–LAD territory (MBF averaged in segments 1,2,7,8,13,14) was lower in cases with total occlusion of the native LAD compared to cases where the LAD was significantly stenosed but not completely occluded (mean MBF 1.43 ± 0.39 ml/g/min, vs 1.90 ± 0.58 ml/g/min; p = 0.005) (Fig. 2). A comparison of stress myocardial blood flow between the basal (AHA 1,2), mid (7,8) and apical (13,14) LAD territory segments showed that native LAD chronic total occlusion (CTO) was associated with MBF reductions in basal (1.47 ± 0.44 ml/g/min vs 2.07 ± 0.64; p = 0.002), and mid segments (1.29 ± 0.34 ml/g/min vs 1.75 ± 0.60 ml/g/min; p = 0.006) but not apical segments (1.52 ± 0.49 ml/g/min vs 1.87 ± 0.60; p = 0.057) (Fig. 3).

### Impact of re-processing quantitative perfusion maps with increased arterial contrast delay on myocardial blood flow estimation

Raw data from myocardial stress perfusion were re-processed using a prolonged arterial contrast delay (maximal arterial time delay threshold 5 s) and were compared to the data obtained using a default arterial contrast delay (arterial time delay threshold 2.5 s). Prolonging the arterial contrast delay threshold to 5 s resulted in a small, but statistically significant increase in global myocardial flow at stress (0.05 ml/g/min, IQR 0.02–0.08; p < 0.001) and rest (0.06 ml/g/min, IQR 0.04–0.09; p < 0.001). Similar changes were observed in the LIMA–LAD territory (Table 3). Global MPR was slightly reduced when arterial time delay was increased to 5 s (− 0.06, IQR − 0.11–(− 0.01); p < 0.001), as the extension of arterial time delay resulted in a proportionally greater increase in rest compared to stress flow (MPR = MBF_stress_/MBF_rest_). To further examine whether arterial contrast delay has a greater impact on MBF of territories supplied only by the LIMA graft, a sub-analysis of cases with patent LIMA grafts and CTO of the native LAD was performed. Presence of native LAD CTO did not result in more profound effect of arterial time delay prolongation on the estimated MBF in the LIMA–LAD territory (Table 3).

**Table 3:** Absolute change in MBF and MPR observed by re-processing the perfusion map data using an increased maximum arterial time delay threshold (from 2.5 to 5 s)

All CABG cases (n = 38)						
Myocardial territory	Change in stress MBF (ml/g/min)	p value	Change in rest MBF (ml/g/min)	p value	Change in MPR	p value
Global	0.05 (0.02–0.08)	< 0.001	0.06 (0.04–0.09)	< 0.001	− 0.06 (− 0.11 to (− 0.01))	< 0.001
LIMA–LAD	0.05 (0.01–0.09)	< 0.001	0.05 (0.03–0.08)	< 0.001	− 0.05 (− 0.10 to 0.00)	< 0.001
**Sub-analysis of CABG cases with totally occluded native LAD (n = 20)**						
LIMA–LAD	0.06 (0.00–0.09)	< 0.001	0.05 (0.03–0.11)	< 0.001	− 0.03 (− 0.08 to 0.00)	0.063
Healthy subjects (n = 25)						
Global	0.01 (0.00–0.03)	< 0.001	0.09 (0.06–0.11)	< 0.001	− 0.25 (− 0.35 to (− 0.20))	< 0.001
LAD	0.01 (0.00–0.01)	0.001	0.06 (0.05–0.09)	< 0.001	− 0.18 (− 0.26 to (− 0.12))	< 0.001

Results shown as median and interquartile range

To evaluate whether the observed differences in MBF caused by study re-processing using a longer arterial time delay were likely to be dependent of the presence of a LIMA graft, 25 healthy subjects perfusion scans were also reprocessed after extending arterial time delay from 2.5 to 5 s. Baseline characteristics and CMR parameters of healthy subjects are shown in Table 1. Healthy subjects also had a minimal but statistically significant increase in global MBF at both stress (median 0.01 ml/g/min, IQR 0.00–0.03, p < 0.001) and rest (median 0.09 ml/g/min, IQR 0.06–0.11, p < 0.001) (Table 3).

The average arterial time delay selected from the MBF estimation algorithm within the LIMA–LAD (in CABG patients) or LAD territories (in healthy subjects) was also assessed (Additional file 1: Figure S2). At stress, the selected arterial time delay (arterial time delay, seconds) was longer for patients with LIMA grafts (1.70 s, IQR 1.20–1.91) compared to healthy subjects (0.71 s, IQR 0.62–0.87; p < 0.001). Similarly, extending the arterial time delay from 2.5 to 5 s resulted in a higher absolute (0.01 ml/g/min, IQR 0.00–0.01 vs 0.05 ml/g/min, IQR 0.01–0.09, p < 0.001) and percentage (0.2%, IQR 0.02–0.67 vs 3.4%, IQR 0.53–5.94; p < 0.001) increase in stress MBF in patients with LIMA grafts compared to healthy subjects (Additional file 1: Figure S2).

## Discussion

This study confirms that perfusion defects in the LAD territory are common in patients referred for perfusion CMR despite LIMA to LAD graft patency and no infarction, and that these defects are predominantly located in the basal and mid rather than apical segments and are associated with native vessel CTO. Finally, the arterial time delay may be longer after LIMA grafting (reflecting arterial transit time) resulting in slight MBF underestimation, but this is not sufficient to explain the degree of flow reduction and hence the perfusion defects. Together these findings suggest that LAD territory inducible perfusion abnormalities are commonly seen in patients with LIMA grafts and are largely due to ongoing abnormalities of myocardial perfusion related to proximal native LAD disease, rather than due to technical limitations related to contrast delay associated with grafts.

Ischemia evaluation in patients with prior CABG is challenging. The complexity of coronary anatomy, the presence of competitive flow, well-developed collateral systems, retrograde blood flow and prior infarction impacting blood flow ascertainment complicate both the interpretation of functional tests and subsequent revascularisation decisions. Previous studies using positron emission tomography (PET) [15], single photon emission computed tomography SPECT [16] and CMR perfusion [5, 17] in patients post CABG, deployed either qualitative or semiquantitative methods to evaluate the impact of coronary graft physiology on MBF. Compared to patients with native vessel coronaries, these studies reported reduced diagnostic performance in patients with grafts [5, 17]. Indeed, patients with prior CABG surgery were excluded from large trials evaluating the diagnostic accuracy of perfusion CMR [18]. Recently, stress CMR using visual (qualitative) assessment demonstrated good discriminative prognostic value in patients after CABG, albeit in a cohort of largely asymptomatic patients [19].

### Myocardial perfusion mapping and underlying coronary anatomy in LIMA graft subtended territories

MBF is governed by processes beyond epicardial coronary flow, including microvascular function [20] and the underlying myocardial architecture. Our data suggest that even in patients with good flow to distal LAD territory via patent LIMA grafts, the main determinant of stress MBF within the LAD subtended myocardial segments was not aspects that affect microvascular function (eg. age, diabetes), but the severity of the underlying native LAD. As one might expect, the impact of native LAD CTO was greatest basally with a gradient, having little effect apically (Fig. 3).

Despite the excellent long term patency of LIMA grafts and their association with improved prognosis, imaging evidence of myocardial ischemia in the LIMA–LAD territory post CABG is not uncommon [21]. Correlation of these functional imaging abnormalities with significant anatomical lesions in the LIMA or distal LAD territories however is variable. In a study using SPECT [22] to evaluate ischemia in the LIMA–LAD territory, half of the patients with detectable ischemia in this territory had no evidence of LIMA graft or anastomosis stenosis on angiography. Importantly, prognosis between those with or without LIMA graft stenosis but perfusion defects in the LIMA–LAD territory was similar. In this study, the authors proposed the mismatch between LAD and LIMA diameters at the anastomotic site as a potential mechanism for their findings, although data on native LAD patency was not provided.

A concern with the use of any first pass perfusion imaging technique in patients with CABG is the increased transit delay in the dynamic contrast delivery to tissue through long graft conduits [23]. Using model-independent deconvolution analysis, Arnold et al. [8], demonstrated that in the context of rest perfusion, despite a short delay associated with contrast arrival and the resulting increased contrast dispersion particularly involving the internal mammary arterial graft, estimation of MBF was not systematically underestimated in graft subtended myocardial territories. Similarly, using semiquantitative perfusion parameters Kelle et al. [9] showed that grafted and native vessel myocardium shared similar contrast kinetics, despite a short delay in contrast arrival in grafted territories.

To address the impact of delayed contrast arrival time on quantitative perfusion, we focused our analysis on a specific model, consisting of patients with a patent LIMA graft to the LAD and with no CMR evidence of myocardial infarction within the LAD territory. We showed that sequence adjustment to accommodate a longer contrast arrival time resulted in a small increase in absolute flow (both at rest and stress), but this could not account for the extent of MBF reduction seen in these territories. Indeed, in agreement with previous semiquantitative data [9], TA prolongation resulted only in a small increase in MBF in the LIMA–LAD myocardial segments (median increase in stress MBF 3.4%). This was similar even in cases where the native LAD was completely occluded, a situation that is theoretically expected to unmask any delay in contrast arrival via the LIMA graft. Importantly, increasing the arterial time delay search window to 5 s would double computational time (as the best arterial time delay value is searched and selected for each myocardial pixel), and is unlikely to result in re-classification of a myocardial segment as having normal MBF.

To further assess whether the increase in MBF observed by extending the allowable arterial time delay was related to the presence of grafts, a healthy subject cohort was used for comparison. Despite a small, but significant increase in the LAD territory MBF in healthy subjects, both the absolute and percentage increase in MBF at stress among healthy subjects was less than the increase observed in patients with grafts following arterial time delay extension (Additional file 1: Figure S2). However, the possibility of unmeasured confounders within the groups contributing to this observation cannot be excluded.

## Limitations

Our study is limited by the relatively small sample size and retrospective nature of data collection. However, the use of an objective artificial intelligence approach to segmentation reduces bias in data analysis. Secondly, although total native LAD occlusion was evaluated in a binary manner to maintain simplicity (present or absent), coronary collaterals were not systematically evaluated. Thirdly, the study focused on myocardial territories usually subtended by the LIMA graft and native LAD where the length of the graft conduit (and hence transit delay) is likely to have most impact, therefore our findings cannot be assumed to apply to other territories. Indeed, differential vasomotor responses between arterial and vein grafts during vasodilator stress was previously proposed [24], but evaluation of this was beyond the scope of this study. Furthermore, variable amount of LGE in territories outside the defined LIMA–LAD segments would make interpretation of MBF in these territories challenging. Importantly, due to the study design, the symptoms prompting the clinical CMR perfusion scan cannot be attributed to the presence of LIMA–LAD territory perfusion defects. It is worth noting that among patients with inducible ischemia in LIMA–LAD territory, only a minority of patients (30%) underwent coronary intervention in a vessel subtending an alternative myocardial territory. Fourthly, our technical analysis specifically considered the arterial time delay of contrast through the LIMA graft conduit, and did not consider additional dispersion or broadening of the arterial input function. Although the effect of long conduits such as the LIMA graft on contrast dispersion was previously thought to be small [8], its impact on absolute MBF estimation warrants further evaluation.

## Conclusion

Perfusion defects in the LIMA–LAD territory are frequently detected with stress CMR imaging in patients with recurrent symptoms post CABG and despite LIMA graft patency. These defects are likely to be the result of persistently low myocardial blood flow (despite patent LIMA grafts) due to native vessel total occlusion, rather than the effect of long graft conduits delaying contrast transit. As expected, this phenomenon is more prominent in the basal rather than apical myocardial segments. Further work correlating findings with invasive physiology and exploring the association with clinical endpoints is required.

## Supplementary Information

**Additional file 1: Figure S1.** Bullseye plot of the left ventricle, demonstrating the American Heart Association model territories used for analysis. **Figure S2.** Top: Comparison of arterial time delay (TA) between healthy subjects and patients with prior CABG. Bottom: Percentage increase in MBF by extending allowable TA to 5 s in healthy subjects and patients with prior CABG. **Table S1.** Predictors of myocardial perfusion reserve (MPR) in the LIMA–LAD territory.

## Data Availability

The datasets used and/or analysed during the current study are available from the corresponding author on reasonable request.

## Abbreviations

AHA: American Heart Association
AIF: Arterial input function
BSA: Body surface area
BTEX: Blood tissue exchange
CABG: Coronary arterial bypass graft surgery
CAD: Coronary artery disease
CMR: Cardiovascular magnetic resonance
CNN: Convolutional neural network
CTO: Chronic total occlusion
LAD: Left anterior descending coronary artery
LGE: Late gadolinium enhancement
LIMA: Left internal mammary artery
LV: Left ventricle/left ventricular
LVEDVI: Left ventricular end-diastolic volume index
LVEF: Left ventricular ejection fraction
LVMI: Left ventricular mass index
MBF: Myocardial blood flow
MPR: Myocardial perfusion reserve
PET: Positron emission tomography
SPECT: Single photon emission computed tomography
