# Supplementary material for: Use of quantitative cardiovascular magnetic resonance myocardial perfusion mapping for characterization of ischemia in patients with left internal mammary coronary artery bypass grafts
Source: J Cardiovasc Magn Reson. 2021 Jun 17;23:82. doi: 10.1186/s12968-021-00763-y (PMC8210347; doi:10.1186/s12968-021-00763-y)
Supplement: Supplementary file 1 — Additional file 1: Figure S1. Bullseye plot of the left ventricle, demonstrating the American Heart Association model territories used for analysis. Figure S2. Top: Comparison of arterial time delay (TA) between healthy subjects and patients with prior CABG. Bottom: Percentage increase in MBF by extending allowable TA to 5 s in healthy subjects and patients with prior CABG. Table S1. Predictors of myocardial perfusion reserve (MPR) in the LIMA–LAD territory. [file 12968_2021_763_MOESM1_ESM.docx]

Additional file (intended for publication as online material)

Figure S1.

Figure S1. Bullseye plot of the left ventricle, demonstrating the American Heart Association model territories used for analysis. Highlighted in red, are the myocardial territories considered to be subtended by the combination of the LIMA graft and native LAD (segments [1,2,7,8,13,14]). For the healthy volunteers, the same territories were thought to be supplied by the native LAD.


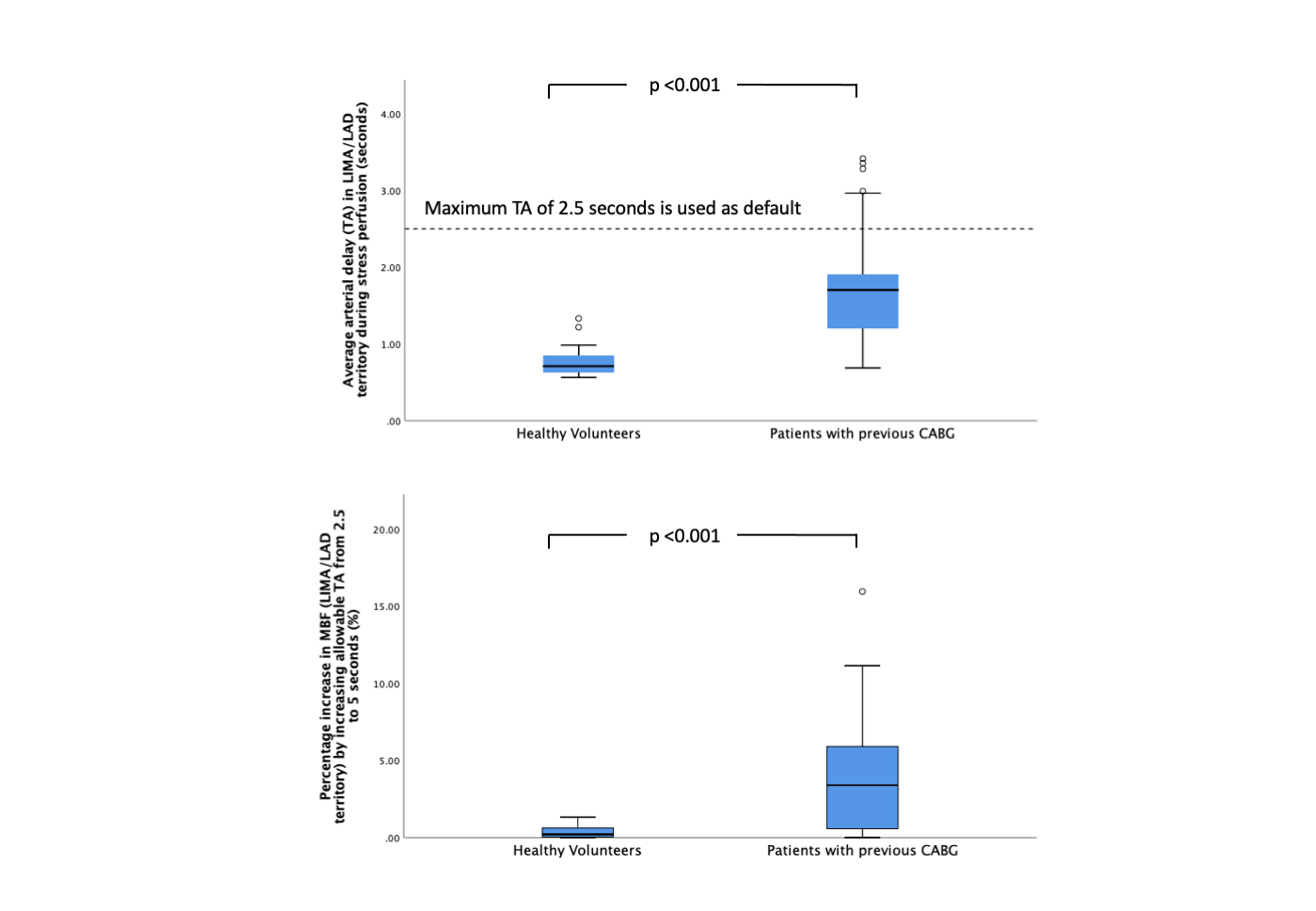


Figure S2. Top: The average arterial delay (TA) within the LIMA/LAD territory (AHA 1,2, 7, 8, 13, 14) was higher in patients with grafts (previous CABG) compared to healthy volunteers (unobstructed native vessels). The TA shown is the average TA within AHA territories (1,2,7,8,13,14). At stress, the selected TA (arterial delay, seconds) was longer for patients with LIMA grafts (median 1.70 seconds, IQR 1.20-1.91) compared to healthy controls (median 0.71 seconds IQR 0.62-0.87; p<0.001). Bottom: The percentage increase in estimated stress MBF in the same territory (AHA 1,2,7,8,13,14) caused by extending the maximum allowable TA from 2.5 to 5 seconds was 0.2% (IQR 0.02-0.67) for healthy volunteers and 3.4% (IQR 0.53-5.94) for patients with grafts (p<0.001).

| Table S1. Predictors of Myocardial Perfusion Reserve (MPR) in the LIMA-LAD territory | | | | | | |
| --- | --- | --- | --- | --- | --- | --- |
|  | **Univariate Predictors** | | | Multivariate Predictors | | |
| Independent variables | B | 95% CI | *P* value | B | 95% CI | *P* value |
| Age | -0.03 | -0.05 - (-0.003) | **0.027** | -0.19 | -0.41 - 0.04 | 0.095 |
| Native LAD occlusion | -0.63 | -1.00 - (-0.25) | **0.002** | -0.56 | -0.95 - (-0.17) | 0.005 |
| LVEF | -0.01 | -0.04 - 0.01 | 0.301 | -0.02 | -0.04 - 0.001 | 0.063 |
| Diabetes | -0.14 | -0.57 - 0.29 | 0.500 | -0.11 | -0.48 - 0.27 | 0.569 |
| LVMi | -0.01 | -0.02 - 0.01 | 0.333 |  | | |
| Sex (Male) | -0.22 | -0.41- 0.86 | 0.476 |  |  |  |
| Beta - blockers | 0.20 | -0.36- 0.76 | 0.475 |  |  |  |
| Bold p values are statistically significant; LAD - Left anterior descending artery; LVEF - left ventricular ejection fraction; LVMi – left ventricular mass index | | | | | | |
